# Solution structure of the *Legionella pneumophila *Mip-rapamycin complex

**DOI:** 10.1186/1472-6807-8-17

**Published:** 2008-03-17

**Authors:** Andreas Ceymann, Martin Horstmann, Philipp Ehses, Kristian Schweimer, Anne-Katrin Paschke, Michael Steinert, Cornelius Faber

**Affiliations:** 1 Department of Experimental Physics 5, University of Würzburg, Würzburg, Germany; 2 Department of Biopolymers, University of Bayreuth, Bayreuth, Germany; 3 Max Planck Research Unit "Enzymology of Protein Folding", Halle, Germany; 4 Institute for Molecular Biology of Infections, University of Würzburg, Würzburg, Germany; 5 Institute Virion\Serion GmbH, Würzburg, Germany; 6 Department of Microbiology, Technical University of Braunschweig, Braunschweig, Germany

## Abstract

**Background:**

*Legionella pneumphila *is the causative agent of Legionnaires' disease. A major virulence factor of the pathogen is the homodimeric surface protein Mip. It shows peptidyl-prolyl cis/trans isomerase activty and is a receptor of FK506 and rapamycin, which both inhibit its enzymatic function. Insight into the binding process may be used for the design of novel Mip inhibitors as potential drugs against Legionnaires' disease.

**Results:**

We have solved the solution structure of free Mip^77–213 ^and the Mip^77–213^-rapamycin complex by NMR spectroscopy. Mip^77–213 ^showed the typical FKBP-fold and only minor rearrangements upon binding of rapamycin. Apart from the configuration of a flexible hairpin loop, which is partly stabilized upon binding, the solution structure confirms the crystal structure. Comparisons to the structures of free FKBP12 and the FKBP12-rapamycin complex suggested an identical binding mode for both proteins.

**Conclusion:**

The structural similarity of the Mip-rapamycin and FKBP12-rapamycin complexes suggests that FKBP12 ligands may be promising starting points for the design of novel Mip inhibitors. The search for a novel drug against Legionnaires' disease may therefore benefit from the large variety of known FKBP12 inhibitors.

## Background

The Gram-negative pathogen *Legionella pneumophila *infects phagocytic cells such as various freshwater protozoa and human alveolar macrophages [1]. The bacteria enter the human lung via aerosols generated by man-made water systems, and cause severe and often fatal human pneumonia particularly in immunocompromised patients. One major virulence factor contributing to infection is the macrophage infectivity potentiator (Mip) protein. *L. pneumophila *strains lacking Mip or expressing a mutant of Mip with low PPIase activity were significantly attenuated in a guinea pig infection model [2]. The protein contributes to the disintegration of lung tissue and subsequent dissemination of the bacteria within the body. Transwell assays support the idea that Mip enables the bacteria to transmigrate across a barrier of lung epithelial cells and extracellular matrix [3].

Mip is a basic 22.8 kDa surface protein (pI 9.8) localized at the outer membrane of the bacteria. Cross-linking experiments revealed that it forms homodimers [4,5]. Mip belongs to the FK506 binding protein (FKBP) family exhibiting peptidyl-prolyl cis/trans isomerase activity (PPIase, EC 5.2.1.8), and is in this respect a homolog of human immunophilins like FKBP12. The crystal structure indicated that each monomer consists of a C-terminal domain, which resembles FKBP12 in its folding pattern and is termed the FK506 binding domain (FKBD). The FKBD is connected via a long (6.5 nm), flexible α-helix to an N-terminal domain which mediates homodimerisation by forming an unusual, symmetrical bundle of four helices with the other monomer [6,7].

**Figure 1:**
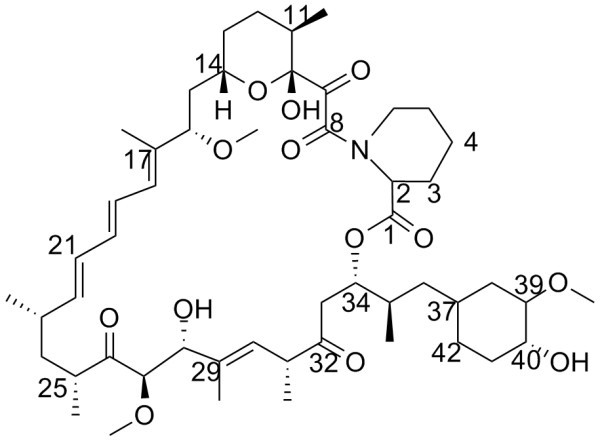
**Structure of rapamycin**. Carbon atoms are numbered.

Although macrolides like azithromycin and chinolones are commonly used and represent efficient antibiotics for treating Legionaires' disease, mortality rates of up to 20% may occur if older or immunocompromised patients are infected. Mip is a potential alternative target for novel antibiotic therapies. The lipophilic macrolides FK506 or rapamycin (Figure 1) both are efficient inhibitors of the PPIase activity of FKBPs, including Mip and FKBP12 [8]. However, these drugs are also immunosuppressive [9,10]. They affect signal transduction pathways for T-cell activation and proliferation by binding to human FKBP12 [11-14], the predominant cytosolic member of the FKBP family. Targets of the emerging complexes are the human proteins calcineurin for FK506 and mTOR, the mammalian target of rapamycin. This in turn affects interleukin-2, which is required for the proper immune response. Hence, neither of the drugs is suitable for the treatment of Legionnaires' disease. A modified ligand blocking specifically the PPIase activity of Mip but lacking the detrimental side-effects on human immune system is a putative agent against Legionnaires' disease. Details of the Mip-rapamycin complex structure would provide insight into the binding processes and would thus allow for the identification of possible modifications of rapamycin to design an inhibitor without side effects. Apart from *L. pneumophila*, FKBP homologues of the Mip sub-family are also present in other human pathogens like *Neisseria gonorrhoeae *[15], *Chlamydia trachomatis *[16] or *Trypanosoma cruzi *[17] making the search for specific ligands even more rewarding [18].

In this article we report on the nuclear magnetic resonance (NMR) investigation of the C-terminal PPIase domain of Mip, comprising about 100 residues with ~35% sequence identity to human FKBP12 [6,19]. Since dimerization is not required for the enzymatic activity we have studied the deletion mutant Mip^77–213^, which only consists of the FKBD with a molecular weight of 14.7 kDa. The solution structures of Mip and of the Mip-rapamycin complex are compared to the FKBP12-rapamycin complex to advance rational design of drugs against Legionnaires' disease.

## Results and discussion

### Structure of Mip^77–213^

**Figure 2:**
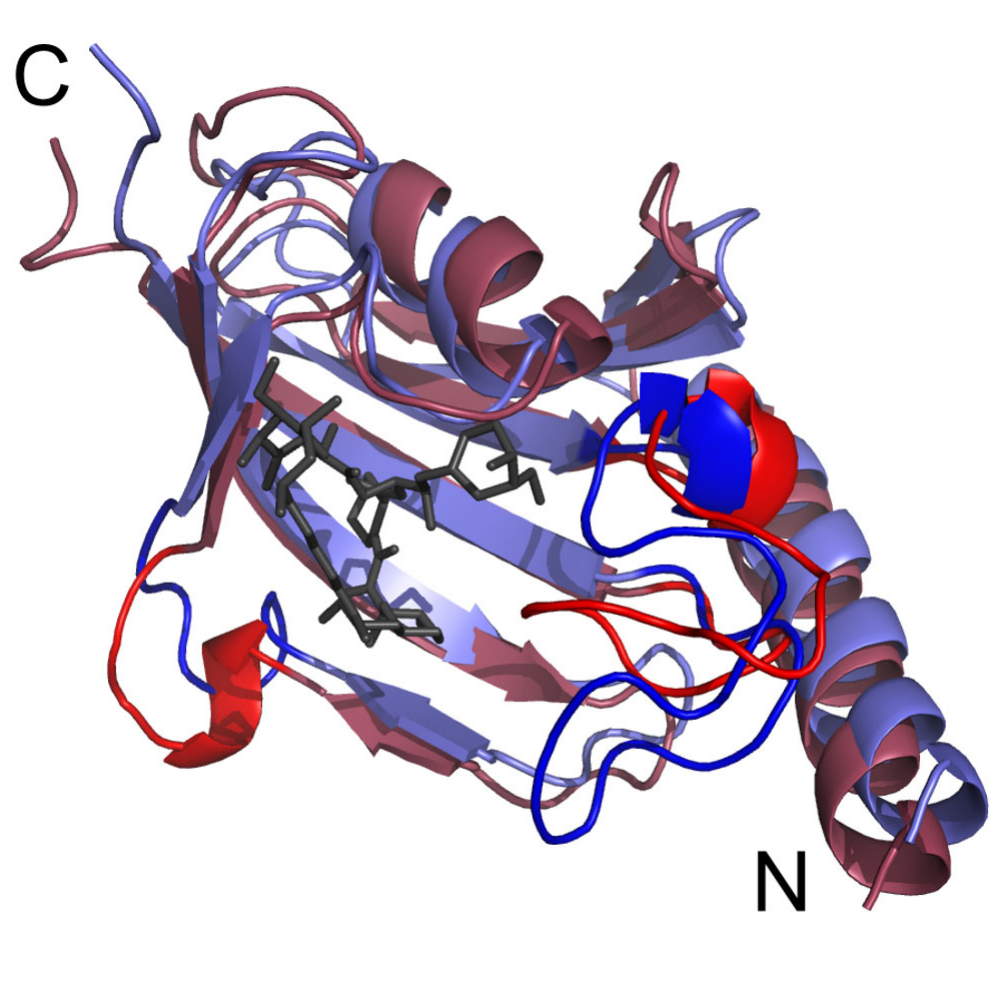
**Overlay of the solution structures of free and rapamycin-bound Mip^77–213^**. The average structures are shown in cartoon representation for free Mip^77–213 ^in red and for Mip^77–213 ^in the complex in blue. Rapamycin is shown in black. The bulge interrupting strand β_4 _(residues T-144 to P-150) and the hairpin loop (residues V-179 to T-199) are highlighted.

Mip^77–213 ^was composed of the C-terminal FKBD and N-terminal amino acids that formed part of the α-helix connecting the two domains in full-length Mip. Statistics for the structure calculation are listed in table 1. The residues N-78 to N-95 of the shortened mutant formed a free-standing α-helix as also observed for the dimer (Figure 2). The C-terminal FKBD, which includes the active site, showed the typical fold, which was nearly identical to the crystal structure of full-length Mip. It consisted of six β-strands, which formed an antiparallel sheet with the topology β_1_-β_2_-β_5_-β_6_-β_3_-β_4_. A short helix α_4 _was located across this sheet. From N-terminus to C-terminus the secondary structure of Mip^77–213 ^included helix _α3 _(N-78 to N-95), strands β_1 _(V-102 to V-103), β_2 _(Q-109 to N-114), β_3 _(T-126 to L-135), β_4 _(separated into two segments comprised of V-140 to S-143 and of A-151 to Q-154 by a bulge of seven residues), helix α_4 _(P-160 to L-166) and strands β_5 _(T-174 to Y-178) and β_6 _(L-200 to V-209). The strands were all connected by short loops except strands β_5 _and β_6 _which were connected by a long hairpin loop (V-179 to T-199). Similar to human FKBP12, a hydrophobic cavity was formed in the presumed center of PPIase activity. It was located between the α_4_-helix and the interior side of the β-sheet and mostly composed of hydrophobic residues. The side chains of W-162 and F-202 formed the bottom of this pocket and were surrounded by Y-131, F-141, D-142, F-153, Q-157, V-158, I-159, P-193, and I-194. The crystal structure of the Mip homodimer is only slightly different from the solution structure presented here. The root mean square deviation (rmsd) between the coordinates of the backbone without the termini (A-81 to V-209) is 0.24 nm between the two structures.

**Table 1:** Structural statistics for free Mip^77–213^

number of structures	10/40
number of restraints	
unambiguous distance restraints	1737
ambiguous distance restraints	784
total distance restraints	2521
dihedral angle restraints	230

rmsd from idealized covalent geometry	

bonds (in nm)	(0.2 ± 0.0) 10^-3^
angles (in deg)	0.4 ± 0.0
impropers (in deg)	0.4 ± 0.0

rmsd from experimental restraints	

distances (in nm)	(2.8 ± 0.3) 10^-3^
dihedral angles (in deg)	1.6 ± 0.1

rmsd values from the minimized average structure	in nm

backbone atoms	0.046 ± 0.010
all heavy atoms	0.085 ± 0.010

Ramachandran analysis	in %

most favored regions	81.8 ± 1.0
additionally allowed regions	16.3 ± 1.1
generously allowed regions	1.7 ± 0.5
disallowed regions	0.2 ± 0.4

### Structure of the Mip^77–213^-rapamycin complex

An analysis of chemical shift perturbation data from the 2D ^15^N-HSQC experiments of free and complexed Mip^77–213 ^indicated significant changes in the chemical environment of residues Y-102, D-142, T-144, F-153 to A-161, A-165, F-185, F-202, and K-203 upon binding of rapamycin. These residues were located in the hydrophobic cavity or in its direct vicinity, clearly indicating that the cleft was involved in binding rapamycin. This had already been assumed from the analysis of the structure of the homologous human FKBP12 in complex with FK506 and rapamycin. The data suggest that the loop between strand β_4 _and helix α_4_, where chemical shift changes were most pronounced, plays a key role in recognition of the ligand.

**Figure 3:**
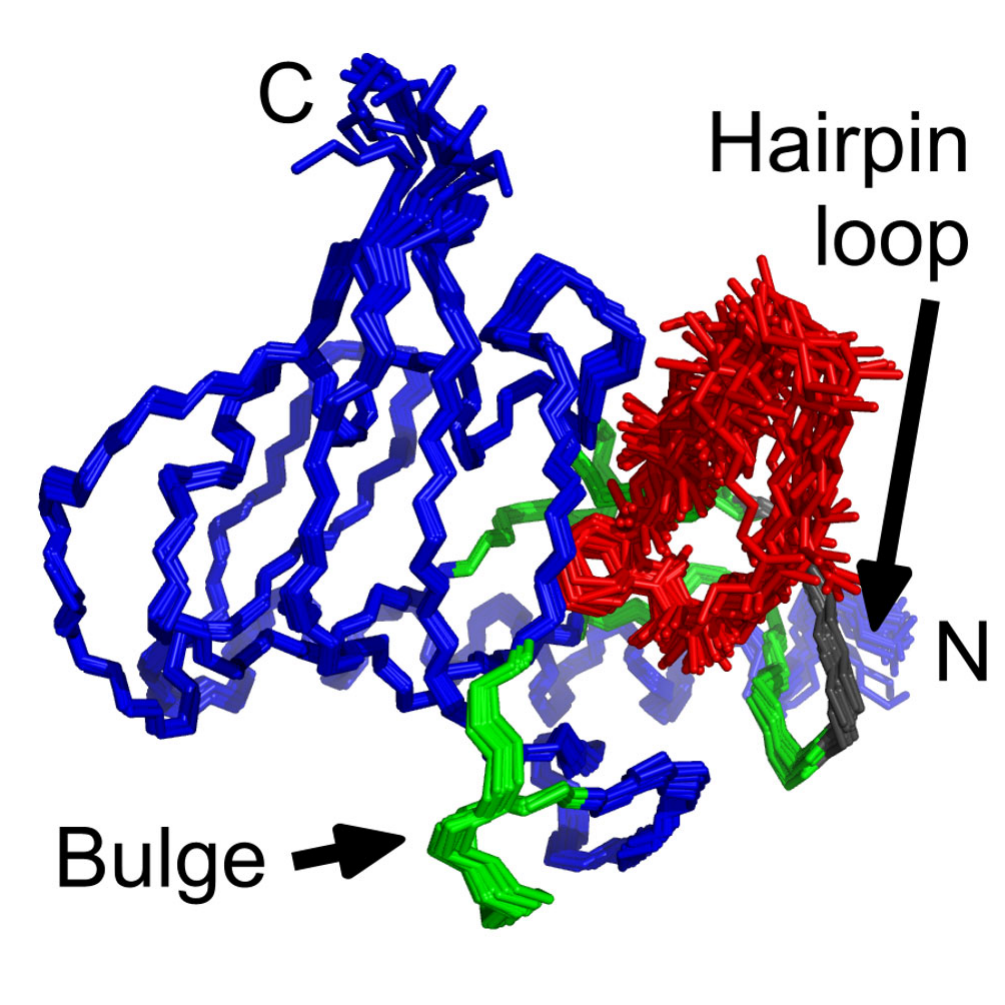
**Ensemble of Mip^77–213^-rapamycin complex structures**. Superposition of the backbone of Mip (blue) and the heavy atoms of rapamycin (red) from the 16 lowest energy structures. The hairpin loop and the bulge are shown in green. The section of the hairpin loop that is stabilized upon binding is indicated in grey.

The average structure of Mip^77–213 ^in complex with rapamycin was similar to the free protein with an rmsd between the backbone coordinates of only 0.26 nm for residues A-81 to V-209 (Figure 2). Structural statistics are listed in table 2. While the secondary structure of Mip remained nearly unchanged, the overlay of the average structures showed a structural rearrangement of the bulge interrupting strand β_4 _(residues T-144 to P-150) as well as of the hairpin loop (residues V-179 to T-199). Upon binding, the former was significantly displaced, enlarging the binding pocket to accommodate the ligand. A distance of 0.60 nm was observed between the positions of G-148 C_α _in the free and bound average structures. Within the hairpin loop, intrinsic changes were observed. The part of the hydrophobic cavity that was formed by P-193 and I-194 in free Mip^77–213 ^became occupied by Y-185, for which a strong change in chemical shift had been observed. The stretch from V-190 to P-196 was bent away from the binding pocket, which was reflected in a lower rmsd of only 0.21 nm between free and complexed protein, if these seven residues were not considered. The hairpin loop and the bulge were the two regions that contributed most to the overall rmsd. Omitting these two regions, the rmsd between free and bound Mip was 0.17 nm for the backbone and 0.15 nm for the secondary structure elements only. The overlay of an ensemble of 16 refined complex structures hinted at flexibility in these two sections. The hairpin loop appeared slightly flexible in the simulations, with a more stable N-terminal part (Figure 3).

**Table 2:** Structural statistics for Mip^77–213^-rapamycin-complex

Number of structures	16/80
Number of restraints	

unambiguous intramolecular distance restraints (Mip^77–213^)	1692
ambiguous intramolecular distance restraints (Mip^77–213^)	2509
intermolecular distance restraints	179
total distance restraints	4380
dihedral angle restraints	230

rmsd from idealized covalent geometry	

bonds (in nm)	(0.7 ± 0.0) 10^-3^
angles (in deg)	0.9 ± 0.0
impropers (in deg)	1.0 ± 0.1

rmsd from experimental restraints	

intramolecular distances (in nm)	(5.6 ± 0.2) 10^-3^
intermolecular distances (in nm)	(21.5 ± 1.8) 10^-3^
dihedral angles (in deg)	2.0 ± 0.0

rmsd values from the minimized average structure	in nm

backbone atoms	0.036 ± 0.009
all heavy atoms	0.081 ± 0.007

Ramachandran analysis (Mip^77–213^)	in %

most favored regions	90.5 ± 1.0
additionally allowed regions	8.0 ± 1.0
generously allowed regions	1.3 ± 0.6
disallowed regions	0.2 ± 0.4

**Figure 4:**
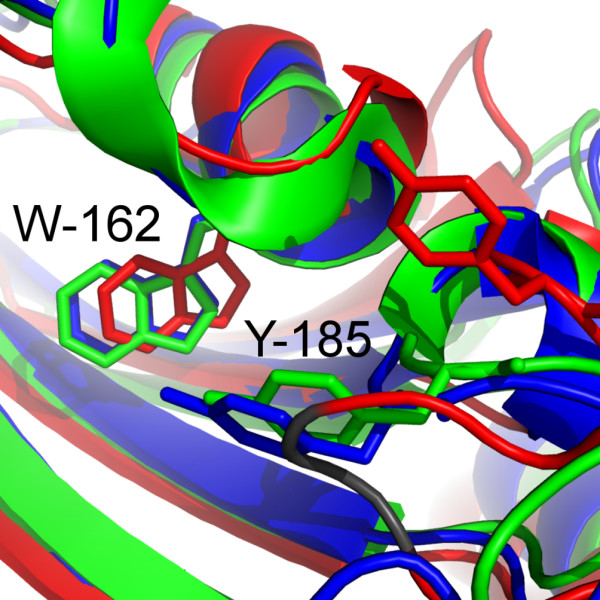
**Detailed view into the hydrophobic cavity of Mip**. W-162 forms the bottom of the cavity. In the solution structure of free Mip (red) Y-185 is not part of the binding pocket. Upon binding (complex structure in blue), the aromatic ring of Y-185 moves into the hydrophobic patch displacing P-193 and I-194, (highlighted in grey) to form contacts with the ligand. In the crystal structure of free Mip (green) Y-185 shows the same orientation as in the complex.

A comparison with the crystal structure revealed that the orientation of the hairpin loop in the Mip^77–213^-rapamycin complex was nearly identical to the orientation in free, full-length Mip. This similar configuration resulted in a backbone rmsd value of 0.15 nm between the two structures for residues A-81 to V-209. In the solution structure of free Mip^77–213^, the orientation of the hairpin loop was different. Y-185, which formed the outer edge of the binding pocket in the crystal structure and in the complex, was displaced, and its position occupied by the residues P-193 and I-194 (Figure 4). This structural rearrangement in Mip^77–213 ^may be an artifact due to the lack of the dimerisation domain. In the crystal structure, the connecting α-helix was stabilized by the hairpin loop via side chain hydrogen bonds withdrawing the residues P-193 and I-194 from the hydrophobic cavity. In the mutant, high flexibility of the N-terminus may have rendered side chain interactions in this part of the helix unfavorable and caused the reorientation of the loop. However, for full-length Mip in solution high flexibility of the hairpin loop was observed by NMR relaxation measurements [7]. Apart from the hairpin loop, all three structures superimposed very well. Without the loop, the rmsd values between the crystal structure and either free or bound Mip were similar (0.17 nm and 0.15 nm, respectively).

**Figure 5:**
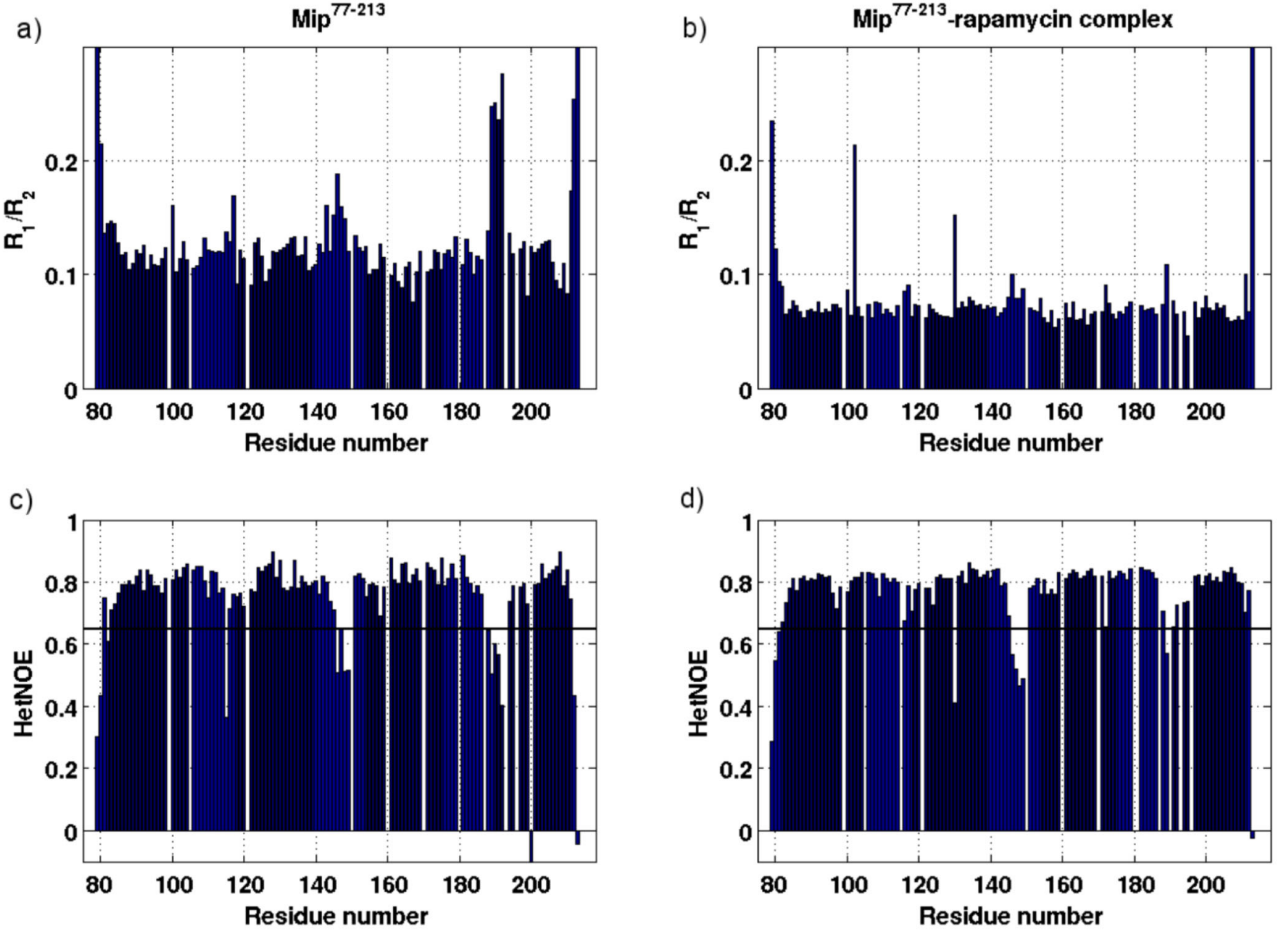
**Relaxation data of free and bound Mip^77–213 ^measured at 14.1 T**. ^15^N-relaxation as measured in a) free Mip^77–213 ^and b) rapamycin-bound Mip^77–213^. HetNOEs are shown in c) for free Mip^77–213 ^and in d) for rapamycin-bound Mip^77–213^. The overall R_1_/R_2 _ratio decreased upon binding of rapamycin, reflecting slower motional tumbling of the complex. Residues with HetNOE values < 0.65 (bold black lines) were not considered for calculation of the correlation time τ_c_. For residues R-188 to G-192 HetNOE values increased and R_1_/R_2 _decreased, suggesting stabilization of these residues in the complex.

To further investigate the stabilization of the hairpin loop, heteronuclear relaxation rate constants R_1 _and R_2 _and Nuclear Overhauser Effects (hetNOE) were measured for rapamycin-bound Mip^77–213 ^and compared to those for the free protein [7] (Figure 5). As had also been observed for free Mip, the relaxation data indicated the presence of a stable secondary structure in the complex. HetNOE values < 0.65, indicating the presence of fast motion on a picosecond timescale [20], were observed for most of the bulge residues (K-146 to K-149) in the complex. This observation provided further evidence for flexibility and fast motion, in accordance with the results of the structure calculations. In free Mip^77–213^, similar values were found for these residues, indicating that the local dynamics of the bulge were not restricted by the presence of rapamycin. Small differences (hetNOE values were slightly lower in the complex) may be due to the structural reorientation of the bulge. Different observations were made for the hairpin loop. For residues R-188 to G-192 of the free enzyme, hetNOEs were smaller than 0.65 and the R_1_/R_2 _values were elevated. Upon binding of rapamycin, NOE values were larger for these residues and R_1_/R_2 _values were not elevated. These differences reflect a decreased flexibility in this part of the hairpin loop in complexed Mip as compared to the free protein. The overall correlation time τ_cee _was derived from the measured R_1_/R_2 _ratios assuming isotropic tumbling. The correlation time was 8.3 ns for free Mip and 11.6 ns for the Mip-rapamycin complex. In order to assess whether this large change is in agreement with the solution structure of the complex, the correlation time for molecular tumbling was calculated from the expected hydrodynamic radius using HYDRONMR [21]. τ_c _values of 9.7 ns and 10.8 ns were obtained for free Mip and the complex, respectively, confirming that molecular tumbling was considerably slowed down by the increased hydrodynamic radius of the complex. The larger increase observed experimentally was most likely due to higher rigidity of the complexed protein, which was not considered in the theoretical model.

Rapamycin was bound to Mip with the pipecolyl ring (C2–N7, see Figure 1 for nomenclature) penetrating deep into the hydrophobic cavity. The ring was surrounded by the aromatic side chains of Y-131, F-153, W-162, and F-202 as well as by residues V-158 and I-159. Intermolecular NOEs were observed for all of these residues except for F-202. The binding domain of rapamycin is comprised of the ester linkage, the pipecolyl ring, the dicarbonyl group, and the pyranosyl ring. The stretch from C14 to C24 was fully exposed, while the cyclohexyl ring was partly accessible to the solvent. Rapamycin was not as well-defined as Mip^77–213^, due to the lack of intramolecular distance restraints. This fact was expressed by a higher average rmsd of the coordinates of all heavy atoms for rapamycin (0.15 ± 0.03 nm) than for the whole complex including the protein (0.081 ± 0.007 nm).

Between Y-185 OH and the rapamycin carbonyl group at C8, an intermolecular hydrogen bond was observed in all of the calculated ensemble structures. Another hydrogen bond involved Y-185 OH and N7. However, there were more possible acceptors for the hydrogen of Y-185 OH at the inner side of the macrolide ring pointing towards the protein in the ensemble. Another hydrogen bond was formed by Y-131 OH and the carbonyl group at C9 of rapamycin. Intermolecular contacts were also found for both oxygen atoms of residue D-142 and the OH-group at C10.

### Sequence conservation

It has been demonstrated by Wintermeyer et al. that both D-142-L and Y-185-A mutations resulted in strongly reduced PPIase activity of the recombinant Mip proteins (5.3 and 0.6% activity compared to wild-type Mip, respectively) [22]. In the complex, both amino acids were observed to be within the hydrophobic cavity and to form hydrogen bonds stabilizing the Mip-rapamycin complex. Since binding of rapamycin efficiently inhibits PPIase activity [8], the hydrophobic cavity of the protein is most likely the active site of the enzyme.

**Figure 6:**
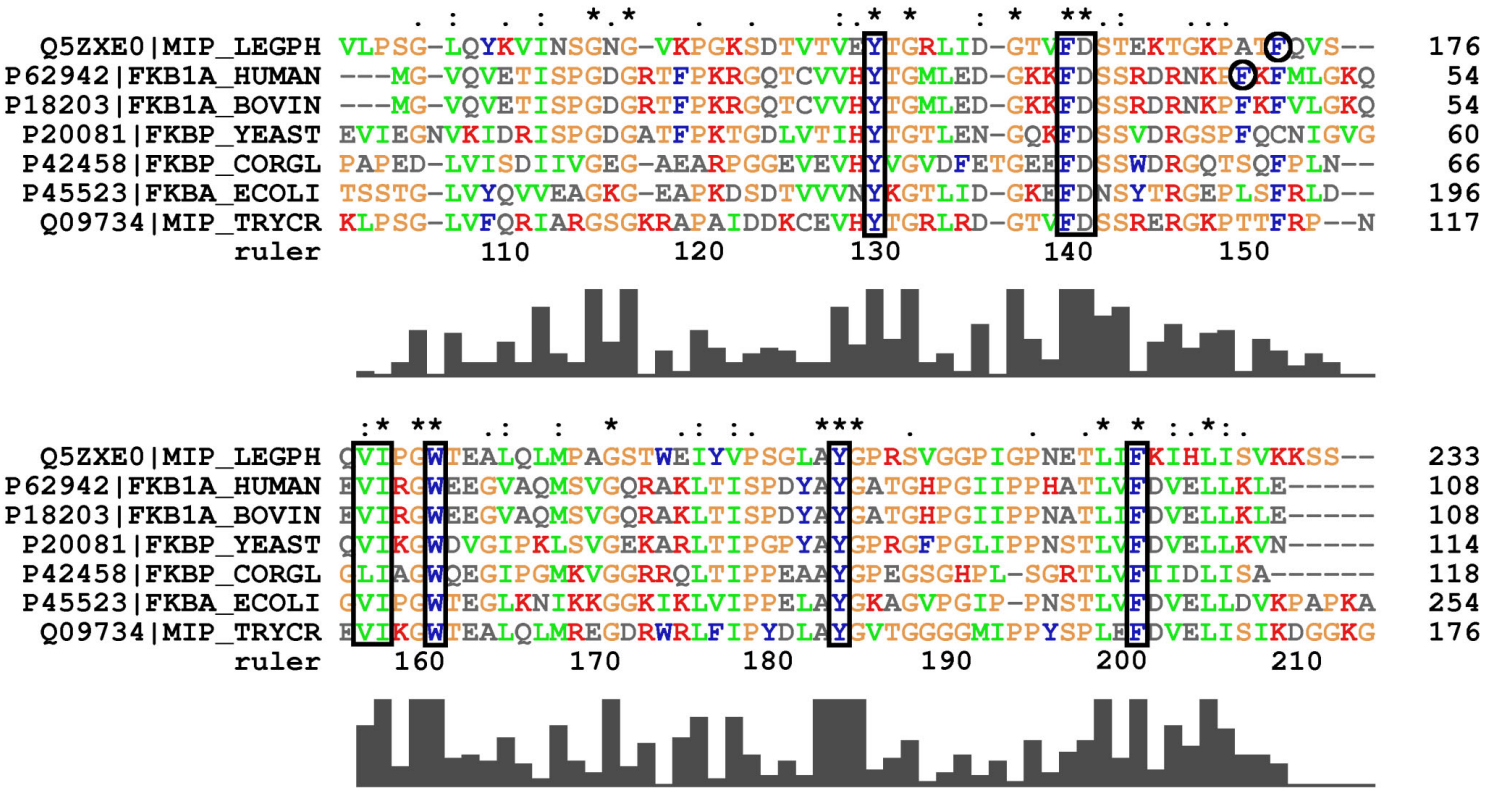
**Multiple sequence alignment of FKBPs and FKBDs from different organisms**. The standard colouring pattern of ClustalX [57] is used. Well and strictly conserved residues that are associated with binding of rapamycin in human FKBP12 and in Mip^77–213 ^are labelled by black boxes. The functional equivalents F-153 in Mip^77–213 ^and F-46 in human FKBP12 (black circles) are neither conserved nor aligned to the same sequence position. Graphical representation of the rate of conservation is indicated at the bottom. Proteins shown: *Legionella pneumophila *Mip, <Q5ZXE0>; *Homo sapiens *FKBP12, <P62942>; *Bos Taurus *FKBP1A, <P18203>; *baker's yeasts *FPR1, <P20081>; *Corynebacterium glutamicum *Cgl0830, <P42458>; *Escherichia coli *FKPA, <P45523>; *Trypanosoma cruzi *Mip, <Q09734>.

The importance of the residues involved in binding of rapamycin is confirmed by their good or strict conservation in species of different kingdoms (Figure 6). Galat has investigated FKBPs with a molecular weight of about 12 kDa and 13 kDa from diverse organisms [23]. The residues corresponding to Y-131, F-141, D-142, F-153, V-158, and W-162 in Mip are well conserved in all sequences among the two groups. I-159, Y-185, and F-202 are even strictly conserved. Except for Y-185, which plays a key role in binding of rapamycin, these residues form the hydrophobic cavity of free Mip^77–213^. Additional residues of Mip involved in binding without being conserved are Q-157, I-194, and especially P-193, which is rarely observed at this position in other FKBDs. The two amino acids of the flexible loop P-193 and I-194 form part of the hydrophobic cavity in free Mip^77–213^, while Q-157 is located on its edge.

### Comparison with human FKBP12-rapamycin complex

**Figure 7:**
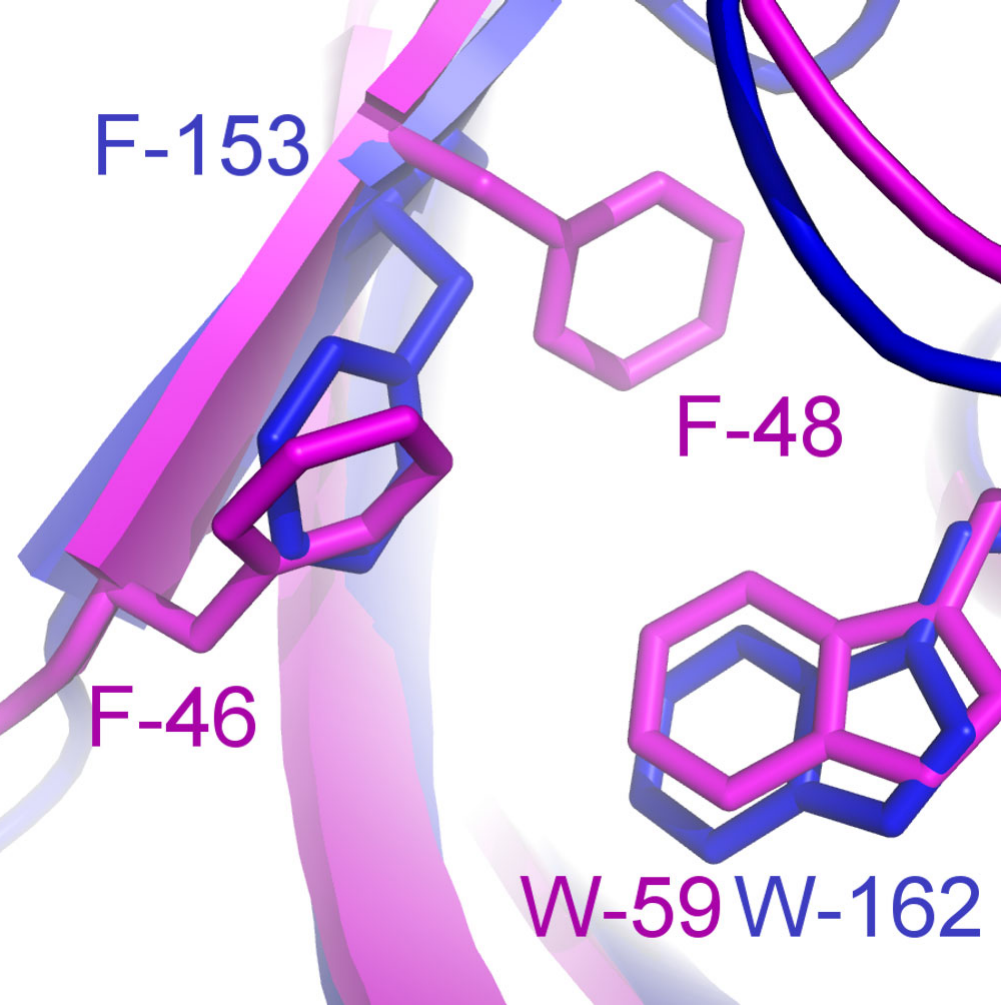
**Detail from the overlay of Mip^77–213 ^and FKBP12 in complex with rapamycin**. Solution structure of the Mip-rapamycin complex is shown in blue and the crystal structure of the FKBP12-rapamycin complex in magenta. In FKBP12, F-48 does not directly contribute to binding of rapamycin. Its analogue in Mip, F-153, takes over the function of F-46 in FKBP12, because residue 151 is alanine in Mip.

**Figure 8:**
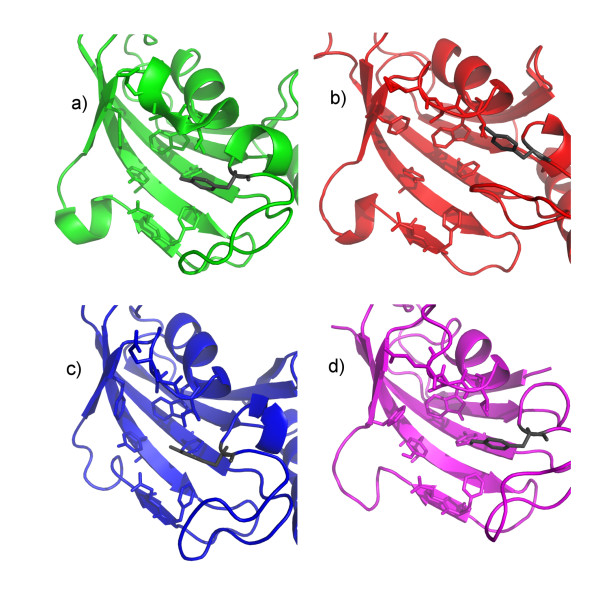
**Topology of the binding pocket in different structures of Mip and FKBP12**. a) Crystal structure of Mip (pdb code 1FD9 [6]); b) solution structure of Mip^77–213 ^(pdb code 2UZ5); c) solution structure of the Mip^77–213^-rapamycin complex (pdb code 2VCD); d) crystal structure of the FKBP12-rapamycin complex (pdb code 1FKB [26]). Side chains forming the binding pocket are shown as sticks. The key residues Y-82 (FKBP12) and Y-185 (Mip) are highlighted in grey in each structure.

The homology of human FKBP12 and *L. pneumophila *Mip is reflected in a high degree of similarity of their hydrophobic cavities. This cavity is formed by the residues Y-26, F-36, D-37, F-46, E-54, V-55, I-56, W-59, Y-82, and F-99 in FKBP12. All residues occupy identical positions as their counterparts in Mip, where F-46 is the only exception. The functional analogue in Mip is F-153, while the corresponding sequence position is occupied by A-151. Interestingly, the sequence position corresponding to F-153 in Mip is occupied by F-48 in FKBP12, which does not directly contribute to binding in the FKBP12-drug complexes. This functional substitution forces a rotation in the side chain of F-153 by about 100° as compared to F-48 in the crystal structure of the FKBP12-rapamycin-complex (Figure 7). The orientation of F153 was experimentally well defined by a total of 73 intramolecular (non-intraresidual) NOEs and 42 intermolecular NOEs to rapamycin. Apart from FKBP12, there are other FKBDs with a triad -FXF- at this sequence position, which is either substituted by -XXF- or -FXX- among other representatives of this group of proteins (Figure 6). This might represent an example of a compensatory mutation [24] during the evolution of the FKBDs. Interestingly, the conformation of the side chain of F-48 in the FKBP12-rapamycin complex is similar to that of one of F-153 in the solution structure of free Mip^77–213^, while the side chain conformation of F-153 in the Mip^77–213^-rapamycin complex is similar to the crystal structure of free Mip (Figure 8). For the secondary structure of bound Mip this has the consequence that the C-terminal segment of strand β_4 _is slightly steeper than in free Mip or in bound FKBP12 (Figure 8). This difference explains the observed displacement of the bulge of sheet β_4_. A further structural difference between the two proteins is that Y-82 forms part of the hydrophobic cavity in both the crystal [25] and the solution structure [26] of free FKBP12. The respective counterpart in Mip is Y-185, which is part of the binding pocket only in the crystal structure, and replaced by P-193 and I-194 in the solution structure. As a consequence, structural rearrangements upon binding with rapamycin are less pronounced in FKBP12 [27,28] than for Mip (Table 3).

**Table 3:** Rmsd values for the hairpin loop between different average structures

Mip (residues V-179 to T-199)	in nm
Solution structure - Crystal structure	0.240
Solution structure - Solution structure of rapamycin complex	0.258
Crystal structure - Solution structure of rapamycin complex	0.151

FKBP12 (residues I-76 to T-96)	in nm

Solution structure - Crystal structure	0.094
Solution structure - Crystal structure of rapamycin complex	0.123
Crystal structure - Crystal structure of rapamycin complex	0.048

Previous NMR investigations of the FKBP12-FK506 [29] complex revealed that, in contrast to the uncomplexed FKBP12, the residues Y-82 to H-87 of the hairpin loop (P-78 to A-95) were rigidly fixed. The flexibility was reduced due to stabilizing interactions by the side chains of H-87 and I-91, as well as by a hydrogen bond from Y-82 to the C8 carbonyl of FK506. The conclusion that Y-82 is a key residue in FKBP12 was further supported by its substitution with leucine [30]. The results for the Mip^77–213^-rapamycin complex are completely analogous to these observations. Y-185, the counterpart of Y-82 in FKBP12, plays the same role in the hairpin loop in Mip. Since this is a common scheme in both proteins, flexibility of the loop may be crucial for the recognition of the protein targets calcineurin and mTOR, respectively, and also for selectivity of the binding.

### Implications for drug design

The high structural similarity between the Mip-rapamycin and FKBP12-rapamycin complexes suggests that FKBP12 ligands are suitable leads for drug design. Rapamycin itself appears as a promising starting point for two reasons. First, rapamycin is an approved and widely used drug, making undesired side effects of its derivatives less probable than for totally new agents. Second, substances based on rapamycin have the potential to be highly active against Legionnaires' disease, because unmodified rapamycin is a subnanomolar inhibitor of FKBP12 [31,32] (K_i _= 0.2 nM) and on the other hand has been shown to inhibit penetration of a lung epithelial barrier by *L. pneumophila *in vitro [3]. The immunosuppresive properties of novel derivatives may be avoided according to the dual domain concept, which implies separate FKBP binding and effector domains in the drug. Immune modulation is mediated by binding a target protein to the effector domain. In the Mip-rapamycin complex molecular contacts are found exclusively to the FKBP binding domain of rapamycin, suggesting that the removal of the effector domain would not influence affinity. Inhibitors composed only of the FKBP binding domain but lacking the effector domain were suggested to have no influence on the immune response. Drug molecules such as, for example, the sub-nanomolar inhibitor V-10,367 (K_i _= 0.5 nM) [33], or a series of sub-micromolar inhibitors of FKBP12 [34], do not affect the immune response or calcineurin activity [35], respectively. However, it has been shown that FK506 (K_i _= 0.6 nM for FKBP12) [31] as well as V-10,367 foster nerve regeneration in SH-SY5Y neuroblastoma cells [36]. These side effects call for further investigations and clinical trials before a novel drug may be approved.

## Conclusion

Structural similarity between the Mip-rapamycin and the FKBP12-rapamycin complexes suggest an identical binding mode of inhibitors in both proteins. The vast number of FKBP12 inhibitors may therefore be used to find a novel agent against Legionnaires' disease. Strategies to avoid unwanted immune modulation caused by interference with calcineurin presumably pertain to Mip as well. Rational drug design starting from known derivatives of rapamycin may take advantage of the presented solution structure of the Mip^77–213^-rapamycin complex. Compared to strategies relying solely on the dual domain concept or bottom-up design this approach potentially offers a better chance to avoid unwanted side effects.

## Methods

### Sample preparation

*Escherichia coli *harboring a plasmid encoding the PPIase domain of Mip and part of the connecting α-helix were used to overproduce Mip^77–213 ^in ^13^C- and ^15^N-labeled medium (Martek M9). The enzyme was purified from these bacteria as described previously [2]. The NMR sample contained 2.5 mM of double labeled (98% ^13^C, 98% ^15^N) Mip^77–213 ^dissolved in 20 mM potassium phosphate buffer in 90% H_2_O and 10% D_2_O at pH 6.5. For the experiments with the complex, unlabeled, dry rapamycin (LC Laboratories, Woburn, USA) was added to the protein solution until the non soluble drug precipitated. The solution containing a 1:1 complex of rapamycin and Mip^77–213 ^was then centrifuged.

### NMR Experiments

The NMR experiments were performed on Bruker Avance 600, 700, and 800 MHz spectrometers at a temperature of 298 K. X-filtered spectra of the complex were acquired on a Bruker Avance 700 with CryoProbe. The data were processed using NMRPipe [37]. Sequence-specific backbone and side chain resonance assignments of free Mip^77–213 ^were obtained as described previously [38]. Assignments for the backbone resonances of rapamycin-bound Mip^77–213 ^were analyzed using 3D HNCO, HNCA, HNCACB, and CBCA(CO)NH spectra. HNHA, HBHA(CO)NH, C(CO)NH and HCCH-TOCSY were used for aliphatic side chain ^1^H and ^13^C assignments. Assignments for the amino groups were obtained by 3D CBCA(CO)NH, 2D HSQC and 3D ^15^N-NOESY spectra. The aromatic ^13^C resonances were assigned from ^15^N-edited NOESY, ^13^C 2D HSQC and 3D NOESY centered at the aromatic frequency. For the assignments of intermolecular distance restraints ^13^C-filtered, ^13^C-edited 3D NOESY spectra were recorded separately for aliphatic and aromatic ^13^C resonances. Both peak picking and visualization of the spectra were performed using NMRView [39].

The ^1^H-^15^N relaxation experiments were performed at 600 MHz, using pulse sequences from Dayie and Wagner [40,41]. The R_1 _values were determined by performing 11 experiments with eight different delays [5.38 (two times), 32.20, 64.38, 128.73 (two times), 300.35, 697.21 (two times), 1297.87, and 1995.06 ms]. To determine the R_2 _values, 11 experiments with five different delays [16.96 (two times, 50.84 (two times), 101.65 (three times), 152.47 (two times), and 203.28 ms (two times)] were performed. For the measurement of the heteronuclear ^1^H-^15^N NOE, the relaxation delay was set to 4 seconds. Proton saturation was achieved by applying 600 high-power pulses with an interpulse delay of 5 ms for the final 3 s of the relaxation delay in the saturation experiment. Correlation times (τ_c_) averaged over different regions of the protein were calculated using the TENSOR2 software package [42,43] assuming isotropic tumbling. Only relaxation rates of residues showing a HetNOE of > 0.65 were used. HYDRONMR [21] was used to estimate the correlation times (τ_c_) from the atomic coordinates files.

### Structure Calculation

Structure calculations were performed on Opteron based multi-core compute servers with 16 GB RAM under Linux. The structure of free Mip^77–213 ^was calculated with ARIA/CNS [44,45] using 1737 unambiguous and 784 ambiguous NOE distance restraints (Table 1). The backbone dihedral angle restraints for the structures of Mip^77–213 ^were determined with TALOS [46]. Calculation of the complex structure was carried out in two steps. First, the structure of rapamycin-bound Mip was calculated with both ARIA/CNS and Xplor-NIH 2.16 [47,48], using 1692 unambiguous and 2509 ambiguous NOE distance restraints (Table 2), which corresponded to 6720 ambiguous atom-to-atom distances in the Xplor-NIH calculations. The backbone rmsd between the average structures obtained with ARIA/CNS and Xplor-NIH was 0.070 nm (0.055 nm for the binding pocket), showing that the different programs introduced only minor deviations compared to the structural differences originating from binding of rapamycin (Table 3).

In the second step, the final average structure of rapamycin-bound Mip was used as input for the calculation of the complex with rapamycin. This significantly decreased the required computing time compared to random structure starting coordinates. Parameters for rapamycin were created by PRODRG2.5 [49] from the crystal structure of its complex with human FKBP12 [27]. To facilitate first assignments of intermolecular NOEs rapamycin was placed manually in the putative binding pocket of Mip. This system was solvated in a periodic box with 10774 SPC water molecules [50] and a 3 ns molecular dynamics run was executed using GROMACS 3.3 [51-53] as described previously [7]. The resulting model was solely used in the first assignment round and not for any further structure calculations.

Complex structures were calculated with Xplor-NIH 2.16 using intra- and intermolecular distance restraints starting with molecules separated by more than 7 nm. The resulting complex structures were compared to the NOEs in the spectra. Reassignment and new calculations were performed in an iterative fashion. In each step, new intermolecular distances were taken into account and the distances violated the most were reassigned or removed. After 14 iterations, 179 intermolecular NOEs were assigned. Refinement of the energetically lowest complex structure after the final run was performed using the modified example script for protein G of Xplor-NIH. Out of the 80 calculated structures, the 16 lowest energy structures were selected for analysis with PyMOL [54] and PROCHECK [55]. All graphical representations of structures were generated using PyMOL.

### Hydrogen bonds

A distance of less than 0.36 nm between donor and acceptor was assumed to be sufficient for an intermolecular hydrogen bond in the Mip^77–213^-rapamycin structures. Thermal noise in the ensemble and the flexibility of residues T-144 to P-150 and A-179 to E-199 caused slight differences in the 16 refined structures. Hydrogen bonds were therefore only accepted if they occurred in at least half of the individual structures.

### Multiple sequence alignment

The sequences were aligned with the ClustalW [56] program and arranged with ClustalX [57].

### Coordinates and chemical shifts

The coordinates of the structures of free Mip^77–213 ^and the Mip^77–213^-rapamycin complex have been deposited at the protein data bank (accession codes 2uz5 and 2vcd, respectively) [58]. Chemical shifts are available at BMRB database (accession code 6334 and 15507, respectively).

## Authors' contributions

AC carried out resonance assignments, calculated the complex structure, analyzed the structures and drafted the manuscript. MH prepared the samples, performed part of the NMR experiments, carried out resonance assignments, and calculated the structure of free Mip^77–213^. PE carried out resonance assignments. KS performed most of the NMR experiments, AKP prepared the protein. MS participated in coordination of the study. CF conceived, designed, and coordinated the study, participated in sample preparation, resonance assignment and drafting of the manuscript, and finalized the manuscript. All authors approved the final version.
